# Renal Denervation for Resistant Hypertension: Time to Improve Patient Selection. The Lesson From ADPKD

**DOI:** 10.3389/fmed.2020.604384

**Published:** 2020-12-18

**Authors:** Antonio Pisani, Carlo Garofalo, Eleonora Riccio

**Affiliations:** ^1^Department of Public Health, Chair of Nephrology, University Federico II of Naples, Naples, Italy; ^2^Nephrology Division, University of Naples-“Luigi Vanvitelli”-Medical School, Naples, Italy; ^3^Institute for Biomedical Research and Innovation, National Research Council of Italy, Palermo, Italy

**Keywords:** renal denervation (RD), ADPKD, hypertension, resistant hypertension, blood pressure

Hypertension is one of the most relevant causes of cardiovascular and renal morbidity and mortality worldwide. About 10% of hypertensive people are not able to reach and maintain optimal blood pressure (BP) values, despite the simultaneous use of 3 antihypertensive agents of different classes at optimal doses, and are considered “resistant”.

The management of resistant hypertension remains problematic: different approaches, including lifestyle modifications and/or a more intensified antihypertensive therapy, have largely failed to improve patients' outcomes and to reduce risk of renal and cardiovascular adverse outcomes.

As the renal nerve plexus plays a central role in regulating arterial blood pressure and renal sympathetic hyperactivity is a major driver of resistant hypertension, minimally-invasive catheter based renal sympathetic ablation (renal denervation) has been proposed as an alternative treatment for resistant hypertension, but the results of clinical study remains ambiguous.

Recently, Blankestijn and Meijvis commented the results of SPYRAL HTN prospective, randomized controlled trial providing evidence of the safety and efficacy of catheter based renal denervation (RD) in unmedicated patients, inserting itself in a long discussion in which hope and disappointment alternated about the safety and efficacy of this method (1). The first results of RD suggested efficacy in treating resistant hypertensive patients, showing a significant reduction in BP.

The Symplicity HTN-1 (2) and Symplicity HTN-2 (3) studies quickly followed, showing dramatic reductions in blood pressure in the systolic range of 30 mmHg compared to a non-blinded control group. These evidences resulted in a growing enthusiasm in the hypertension community with a consequent expanding procedural indications and technology evolution.

However, the results of Symplicity HTN-3, the first randomized sham-controlled trial, failed to demonstrate a significant difference in BP reduction between RD and t sham control group (4).

Despite of the setback following the SYMPLICITY HTN-3 trial and two other sham-controlled, randomized studies, the Symplicity Flex (5) and the RESET (6), more recent sham-controlled trials and last the study by Bohm and colleagues providing proof of principle for the BP-lowering efficacy reaffirming the validity of this technique in the absence of antihypertensive medications (7).

However, the treatment differences observed in SPYRAL HTN study between RD and sham procedure of 3.9 mmHg for 24 h systolic BP and 6.5 for office systolic BP, again raise in Blankestijn the doubt about widespread acceptance of RD as therapy for hypertension in general population. He rightly claims that hypertension is a heterogeneous condition and that it is unrealistic to think that RD could be a panacea for all hypertensive patients. Several issues have to be resolve before RD overcome all the disappointments of recent years and be ready for prime time of hypertension treatment: identification of clinical or biomarker effect predictors, assessment of long-term efficacy, evaluation the effects on cardiovascular and end-organ damage, but most of all the identification of patients with the highest likelihood of response.

At the moment, however, for many of these problems the real solution seems to be far away.

RD efficacy is correlate to an overactivity of sympathetic system in renal nerves. In healthy people, renal nerve activity is usually low and the direct assessment is impossible while the effectiveness of the method procedure evaluation is far from clinical use (8, 9).

Starting from these unsolved issues Blankestijn concludes that is time to refine the focus of research in the renal denervation field. Perhaps, as indicated by the autosomal dominant polycystic kidney disease (ADPKD) lesson, called into question by the same author, more simply is the time first to improve the hypertensive population selection.

Hypertension is common in ADPKD: prevalence is 50to 62% when renal function is still normal and increases to almost 100% in patients with chronic renal failure with fewer than 30% achieve BPs less than 130/80 mmHg despite the treatment (10). The pathogenesis of hypertension in ADPKD patients remains complex and depending on many interrelated factors. Evidences in mice and human with ADPKD, however, showed that sympathetic overactivity plays a crucial pathogenetic role (11). On these basis, few years ago we performed, due to failure of all other therapeutic strategies, including a uninephrectomy, the RD by radiofrequency ablation of the single principal renal artery in a woman with single-kidney stage CKD secondary to ADPKD and uncontrolled treatment-resistant hypertension. After the procedure, the patient's BP declined remarkably, decreasing her need for antihypertensive medications without a significant decline in kidney function (12).

Consistent with this, several case reports have suggested that RD is effective for BP control and able to improve characteristic abdominal pain in ADPKD patients. Without doubt we should learn le lesson of Symplicity trials in RD safety and efficacy evaluation and we are convinced that case reports and uncontrolled studies is not enough. However, the experience in ADPKD it seems to indicate that the correct selection of the patient population, with a preference for those where sympathetic hyperactivity as a key factor in the genesis of hypertension is demonstrated in animal and human models and supported by *in vitro* studies, is the real first step to take for the different RD techniques to become ready for prime time in the hypertension treatment scenario.

## Author Contributions

AP had the original idea. ER and CG collected data. AP, ER, and CG wrote the paper. All authors contributed to the article and approved the submitted version.

## Conflict of Interest

The authors declare that the research was conducted in the absence of any commercial or financial relationships that could be construed as a potential conflict of interest.
